# Health Tract, No. 89

**Published:** 1865

**Authors:** 


HEALTH TRACT, NO. 89.

### LAW OF LOVE.

Said an old man one day: "When I look back over the long pilgrimage of an eventful and not unsuccessful life, I can confidently say that I never did a kindness to any human being without finding myself the happier for it afterward. A single friendly act, cheerfully, pleasantly, and promptly done to a fellow-creature in trouble or difficulty, besides the good to him, has before now thrown a streak of sunshine into my heart for the remainder of the day, which I would not have taken a twenty-dollar bank-note for."

If such acts of thoughtfulness and consideration and humane sympathy were performed as we "have opportunity," the same "streak of sunshine," the same lightening up of the load of life would come to both giver and receiver, until after a while there would be sunshine all the time within us and without, dispersing physical as well as moral miasms, purifying the social and domestic atmosphere, warming the heart to still higher sympathies, and waking up the whole man to those activities which can never fail to preserve, maintain, and perpetuate mental, moral, and physical health, to a serene old age. These things are to be done at home and abroad, at the family table, the fireside, in the street, on the highway, in town, in country, by day and by night, always and every where, kindly and cheerily, whenever there is "opportunity;" to be done to the old and the young, to the rich and the poor, to the sick and the well, to the successful and the unfortunate, to stranger and acquaintance, to man and woman, enemy and friend, to every body and to every thing that breathes the breath of life. These sunlight-giving kindnesses can be done in multitudes of cases by a word, a smile, a look. And these cost so little, why should they not be thrown broadcast over the whole surface of humanity, in princely profusion, blessing as they do the giver as well as receiver, giving gladness to both, and a quiet peace which gold could never purchase, which diamonds of the purest water and gems of richest hue could not secure for the briefest hour? Men, women, children, all, wake up from this good hour, and make the "law of love" to all of human kind the pole-star of life, the work, the pleasure of your human existence; and in that triumphant hour when you shall be called to close your eyes on all things earthly, and open them on the realities of an eternal existence, the first sound that shall fall upon your delighted ear from the heavenly shore, will come from the King in his beauty, when he shall say: "Ye did it unto me. Well done!"

